# Integration of GPT-4 into multimodal bioinformatics for surgical specimens

**DOI:** 10.1097/JS9.0000000000001617

**Published:** 2024-05-15

**Authors:** Siqi Fan, Yue Zheng, Xu Sun, Ailin Zhao, Yijun Wu

**Affiliations:** a Division of Thoracic Tumor Multimodality Treatment, Cancer Center, West China Hospital, Sichuan University; b Department of Hematology, West China Hospital, Sichuan University; c Laboratory of Clinical Cell Therapy, West China Hospital, Sichuan University; d West China School of Medicine, Sichuan University, Chengdu, Sichuan, People’s Republic of China

*Dear Editor,*

In recent years, the development of multimodal deep learning has made the application of large multimodal models (LMMs) in image processing and surgical diagnosis possible, and we note that recently Zhu *et al*.^1^ have offered an interesting insight into GPT-4’s potential application in the interpretation of radiological images and decision making. The authors have evaluated GPT-4’s performance in making diagnoses and further treatment plans by discerning images attached with patient histories. Besides, this article also compared the diagnostic accuracy of the GPT-4 model when provided with and without patient histories. Thirdly, it assessed GPT-4’s capability of managing complicated cases. GPT-4’s commendable performance in analyzing radiological images indicates LMMs’ exciting potential for future development and application in surgery. Based on this, we’d like to further discuss the multimodal GPT-4’s possible application of assisting bioinformatics research for surgical specimens.

GPT-4 may serve as a user-friendly interface for surgical teams seeking to harness LMMs for bioinformatics research. Models undergoing fine-tuning for specialized biology-centric endeavors, encompassing text-based analysis, biological sequence decoding, and database classification, have a big role in such research. Noteworthy contributions include expediting diverse bioinformatic activities, ranging from literature review to data analysis, result interpretation, and visualization. Furthermore, it facilitates surgical teams through accessible code interpreters and plugins. Despite the potency and revolutionary nature of GPT-4, it remains an incipient tool that is susceptible to yielding inaccurate results and lacks algorithmic transparency, reliability, and security because of the inherent constraints of LMMs based on experience and data perception (Fig. 1A). In addition, because of the absence of comprehensive background information and ethical considerations, bioinformatics applications have not been as desirable. These drawbacks underscore human guidance, creative thinking, and censorship when using artificial intelligence (AI)-based platforms to enhance research progress.

**Figure 1:**
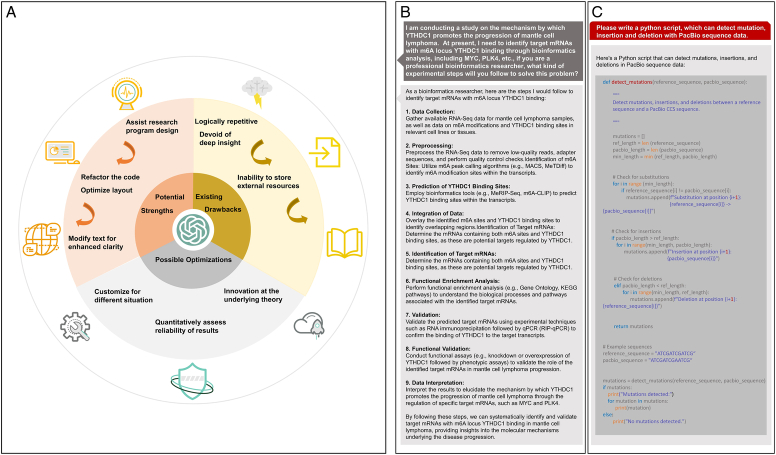
The role of GPT in multimodality bioinformatics research. (A) ChatGPT in bioinformatics research. (B) Example of applying ChatGPT to design a bioinformatics research program. (C) Tests of ChatGPT in aiding data analysis.

Data mining and literature reviews can be arduous, particularly when extensive genes must be filtered. Additionally, scrutinizing candidate genes via literature review can be labor-intensive, with inherent limitations from constrained scope and human biases, thereby compromising validity and reliability. GPT-4 demonstrates proficiency in processing substantial volumes of information through natural language instructions and imported datasets swiftly and effectively. Therefore, it can be applied to design bioinformatics research programs (Fig. 1B). Additionally, its capacity for training with base models and usage patterns enables virtual screening for molecular modeling and cell-type annotation^2^. For example, BioMedGPT, an open-source model, has amalgamated diverse data sources such as genes, molecules, cells, proteins, literature, patents, and knowledge bases. Such refinement augments the relevance and utility of the data provided by GPT, thereby enhancing the efficiency and efficacy of the screening process.

The integration of the National Center for Biotechnology Information interface with GPT-4 facilitates the execution of bioinformatic analysis tasks, especially for surgical specimens^3^. We conducted tests to assess coding capabilities, such as mRNA polyadenylation length calculation and mutation detection, all of which were accomplished through natural language interactions (Fig. 1C). The interaction capacity of ChatGPT enhances coding efficiency while ensuring conciseness. It effectively optimizes code operations by adjusting loops to conserve memory and reorganizing repetitive segments to improve clarity through function extraction. Moreover, GPT-4 demonstrates proficiency in executing intricate tasks involving large volumes of biological data, particularly in time-sensitive biomedical processing tasks.

Another noteworthy advantage of bioinformatics novices lies in GPT-4’s ability to append explanatory comments to code and rename variables using an accessible code interpreter and plugins. This significantly reduces redundancy and facilitates efficient modifications and additions. Furthermore, in the context of debugging, GPT-4 not only identifies errors but also provides precise feedback on their origins and suggests corrected lines of code. The primary utility of ChatGPT lies in its support for writing tasks, extending beyond rectifying grammatical errors to generating language that is comprehensible to both biologists and computer scientists, aiding non-native English speakers. Users can prompt variations in their expressions and select their preferred versions, thereby enhancing their flexibility. Moreover, GPT-4 proves advantageous in tailoring text for diverse audiences, including crafting media releases and simplifying research for non-specialists. It is essential to recognize that when utilizing AI-generated text, clarity in declarations is imperative to prevent misunderstandings or breaches of ethical requirements. Furthermore, GPT-4’s role in optimizing data visualization is notable, as it proficiently generates code for ggplot2 and ggcharts packages in R, as well as Matplotlib, thereby alleviating the burden of memorizing an array of complex drawing commands to some extent. GPT-4 interprets the drawing code and generates the corresponding code based on the examples provided. In addition, GPT-4 aids in color selection for diagrams, improves accessibility for individuals with color vision deficiencies, and offers suggestions for enhancing the visual layout.

Despite exhibiting advanced proficiency in understanding and reasoning about bioinformatics, concerns persist regarding the reliability of ChatGPT. These concerns stem from the potential for misinterpretation or incorporation of erroneous information during the generation of code or textual content, leading to potential inaccuracies. To mitigate these risks, it is suggested that users provide more detailed information and specific goals, thereby increasing the likelihood of GPT-4 to generate informative responses. Additionally, it is crucial to acknowledge that despite its capacity for multiple rounds of optimization, prolonged and intricate conversations pose an elevated risk of context loss. Besides, the concerns associated with ChatGPT in supplementary scientific research primarily encompass information distortion, data breaches, unaware plagiarism and infringement, and scientific research immorality. AI tools, such as ChatGPT, have become part of the bioinformatics realm; however, ChatGPT is more of an exploratory tool than a reliable tool in scientific research.

Several teams are currently involved in refining LMMs specifically designed for medical and surgical applications, as evidenced by initiatives such as ChatDoctor, HuaTuo, and PMC-LLaMA^4,5^. These efforts focus on medical question-and-answer scenarios and have shown promising results. However, the lack of a comprehensive consideration of the broader clinical context within ChatGPT’s analytical framework poses a challenge. From this perspective, its functional efficacy appears to be inferior to that of other bioinformatics tools. Moreover, the absence of an option to install external packages and the inability to store external resources exacerbate this issue, presenting barriers to its widespread adoption in bioinformatics applications. Achieving a sufficiently intelligent and professionally adept ChatGPT requires prolonged effort to accumulate the necessary data and refine its capabilities accordingly.

The future role of ChatGPT is envisioned as a research-oriented personal assistant, streamlining research tasks and allowing researchers to focus more intently on overarching objectives. Specifically, ChatGPT is expected to aid in formulating protocols for the model evaluation and validation of classification outcomes. It will also facilitate inter-process and cross-platform operations, as well as result reproducibility, enabling the incorporation and storage of external resources and result verification. Additionally, ChatGPT is anticipated to assist in fine-tuning learning models and enhance the precision and efficacy of bioinformatics research. Recent developments include the release of ChatGPT’s open-source alternative, Claude 3, and efforts in natural language embedded program development, which offers a structured language-generation framework. This evolution in design aims to provide one-click solutions to natural language, mathematical reasoning, symbolic reasoning, and programming problems in a transparent, controllable, and accurate manner.

In conclusion, ChatGPT and multimodal GPT-4 introduce novel prospects for multimodal bioinformatics for surgical specimens, promising to alleviate burdens and provide innovative, efficient, and optimized solutions for bioinformatics research and education. Despite these affirmative aspects, challenges persist. The inherent shortcomings include interpretability and reliability deficiencies, the generation of inaccurate responses, and susceptibility to context loss, necessitating innovative alterations at the foundational theoretical level. Moreover, the ethical dimensions of this evolution must be rigorously addressed; in the era of AI, contemplation of the concept of originality and the delineation thereof becomes pertinent. In addition, the development of practical and deployable methodologies for the quantitative assessment of result reliability, such as furnishing corresponding confidence levels with output results, can mitigate the trust crisis in deep learning.

## Ethical approval

Not applicable.

## Sources of funding

This work was supported by the Postdoctoral Fellowship Program of CPSF (No. GZB20230481), National Natural Science Foundation of China (No. 82303773, No. 82204490), Natural Science Foundation of Sichuan Province (No. 2023NSFSC1885), and Key Research and Development Program of Sichuan Province (No. 23ZDYF2836).

## Author contribution

All authors read and approved the final version of the manuscript.

## Conflicts of interest disclosure

There are no conflicts of interest.

## Research registration unique identifying number (UIN)

Not applicable.

## Guarantor

All the authors of this paper accept full responsibility for the work and/or the conduct of the study, have access to the data, and control the decision to publish.

## Data availability statement

No primary data were generated and reported in this manuscript. Therefore, data have not become available to any academic repository.
